# Protective Effect of Topiramate on Hyperglycemia-Induced Cerebral Oxidative Stress, Pericyte Loss and Learning Behavior in Diabetic Mice

**Published:** 2015-03-16

**Authors:** Tulin O. Price, Susan A. Farr, Michael L. Niehoff, Nuran Ercal, John E. Morley, Gul N. Shah

**Affiliations:** 1 Division of Endocrinology, Department of Internal Medicine, Saint Louis University School of Medicine, St. Louis, MO, USA; 2 Research and Development, St. Louis Veterans Affairs Medical Center, St. Louis, MO, USA; 3 Division of Geriatric Medicine, Saint Louis University School of Medicine, St. Louis, MO, USA; 4 Department of Chemistry, Missouri University of Science and Technology, Rolla, MO, USA

**Keywords:** Diabetes, Brain microvasculature, Cognition, Topiramate, Oxidative stress, Mitochondrial CAs

## Abstract

Diabetes mellitus-associated damage to the microvasculature of the brain is caused by hyperglycemia-induced oxidative stress, which results in pericyte loss, blood-brain barrier disruption, and impaired cognitive function. Oxidative stress, in diabetes, is caused by reactive oxygen species produced during accelerated respiration (mitochondrial oxidative metabolism of glucose). The rate of respiration is regulated by mitochondrial carbonic anhydrases (CAs). Inhibition of these enzymes protects the brain from diabetic damage. Previously, we reported that topiramate, a mitochondrial CA inhibitor, at a dose of 50 mg/kg/day protects the brain in diabetes by reducing oxidative stress and restoring pericyte numbers. Topiramate has high affinity for both mitochondrial CAs; therefore, it is conceivable that a much lower dose may inhibit these enzymes and thus protect the brain from hyperglycemia-induced oxidative damage. Therefore, in an effort to reduce the toxicity associated with higher doses of topiramate, the current study was designed to investigate the effect of 1.0 mg/kg topiramate on reducing oxidative stress, restoring pericyte numbers in the brain, and improving the impaired learning behavior in diabetic mouse. Diabetes was induced by a one-time injection of streptozotocin and topiramate was administered daily for 12 weeks. Levels of oxidative stress, reduced glutathione (GSH) and 4-hydroxy-2-trans-nonenal (HNE) were measured in the brain and pericyte/endothelial cell ratios in isolated brain microvessels. Learning behavior was assessed by T-maze foot shock avoidance test. A significant decrease in GSH (control, 12.2 ± 0.4 vs. diabetic, 10.8 ± 0.4 vs. diabetic + topiramate, 12.6 ± 0.6, p<0.05) and an increase in HNE (control, 100 ± 4.2, vs. diabetic, 127.3 ± 8.8 vs. diabetic + topiramate, 93.9 ± 8.4 p<0.05) in diabetic mice were corrected by topiramate treatment. Topiramate treatment also resulted in restoration of pericyte numbers in diabetic mice (control, 25.89 ± 0.85 vs. diabetic, 18.14 ± 0.66 vs. diabetic + topiramate, 24.35 ± 0.53, p<0.001) and improvement in learning behavior. In conclusion, these data clearly demonstrate that topiramate at 1.0 mg/kg protects the mouse brain from diabetic damage. A 1.0 mg/kg topiramate in the mouse translates to a 5.0 mg daily dose in a 60 kg human, which may help slow the onset and progression of diabetic complications in the human brain.

## Introduction

Diabetes mellitus, a chronic metabolic disorder, is associated with hyperglycemia, which causes a significant increase in oxidative stress, resulting in damage to the microvasculature of brain and subsequent cognitive impairment [1–7]. Hyperglycemia-induced oxidative stress plays a key role in the development and progression of complications associated with diabetes in the brain, including pericyte loss [8], disruption of the blood-brain barrier (BBB) [9,10], and learning and memory deficits [11]. Proper functioning of the brain microvasculature is critical for maintaining optimal brain function. The brain parenchyma is separated from blood by the BBB, which is a complex feature of brain endothelial cells (BEC). The BBB restricts the passage of blood-borne molecules and facilitates delivery of essential nutrients and selected biomolecules into the brain parenchyma. Although the vascular BBB is physically made up of BEC, pericytes in close contact and interaction with BEC in the microvasculature are vital for development and integrity of the BBB as well as its proper function [12–14]. Pericytes are in the first line of defense against the deleterious effects of oxidative stress. Loss of pericytes underlies disruption of the BBB in Alzheimer’s disease [15,16] and in diabetic retinopathy [17,18], where the blood-retinal barrier is an extension of the BBB [19]. We have shown that the diabetic mouse brain exhibits oxidative stress and loss of pericytes twelve weeks after the onset of diabetes [8] and that high glucose-induced intracellular oxidative stress causes pericyte death by apoptosis in culture [20].

Several studies have shown an association between diabetes and memory deficits [21,22]. Diabetes-induced microvasculature changes in the brain were shown to be predictive of cognitive deficits in patients [23]. Diabetes is associated with lower cognitive function and higher incidence of dementia, including Alzheimer’s disease and vascular dementia that leads to memory dysfunction [24]. Moreover, hyperglycemia-induced oxidative stress triggers degenerative events in the hippocampus (a critical brain region for learning and memory) that lead to memory dysfunction in diabetes [25,26].

Previously, we reported that 50 mg/kg/day of topiramate protect the mouse brain from diabetic damage [8]. The current study was designed to evaluate the effect of a lower dose (1.0 mg/kg/day) of topiramate on diabetic damage to the brain as well as learning dysfunction in mice, in an effort to reduce the toxicity associated with the higher doses of topiramate [27].

We now report that a low dose of topiramate (1.0 mg/kg/day) reduces hyperglycemia-induced oxidative stress and restores pericyte numbers. Moreover, this same dose improves the learning behavior that was impaired by diabetes in these mice. The 1.0 mg/kg/day in the mouse is equivalent to 5.0 mg/day [28] in a 60 kg human, which is forty times less than the average clinical dose currently in use for other diseases. These findings suggest that topiramate at 5.0 mg/day may protect the human brain from diabetic damage caused by hyperglycemia-induced oxidative stress and will be better tolerated by patients due to fewer side effects compared to higher doses currently in clinical use for other diseases.

## Materials and Methods

### Animals and Experimental Protocols

Male CD-1 mice from an in-house colony, 8 weeks of age, served as test subjects. This colony has been maintained as an outbred strain obtained from Charles Rivers Breeding Laboratories of Wilmington, MA, USA. The mice are tested regularly to ensure that they are virus and pathogen free. All subjects were experimentally naïve. Mice were on a 12 h light:12 h dark cycle with lights on at 0600. Food (PMI Nutrition LabDiet 5001, LabDiet, St. Louis, MO, USA) and citrate water (1.68 mM sodium citrate, 1.57 mM potassium citrate; C7254, C8385, respectively) were available ad libitum. Citrate water helps improve the survival of streptozotocin (STZ)-diabetic mice [29]. All experiments were conducted after approval by the Saint Louis University Institutional Animal Care and Use Committee, which abides by the NIH Guide for Care and Use of Laboratory Animals.

### Drugs and Treatment

Topiramate and STZ were purchased from Sigma-Aldrich (St. Louis, MO, USA). The anti-HNE was obtained from Alpha Diagnostics (San Antonio, TX, USA). Gamma Tubulin was from ThermoFisher Scientific (Rockford, IL, USA).

Eight-week-old male CD-1 mice were rendered diabetic by a one-time intravenous tail vein injection of freshly prepared 150 mg/kg STZ in vehicle (0.2 ml of 0.9% NaCl with 2 mg/ml citric acid, pH 4.5) according to the standard method [30]. Control mice received injections of vehicle. Diabetes was verified 48 hours after STZ injections by measuring tail vein blood glucose with a Glucometer (ReliOn, Arkray, MN, USA). Only animals with glucose levels ≥250 mg/dl [8] were considered diabetic and included in the study. Two days after STZ or vehicle injection, mice were divided into the following groups (n=10 in each group): (i) non-diabetic (controls); (ii) diabetic (DM); and (iii) diabetic mice injected with topiramate (1.0 mg/kg) (DM+TOP).

Topiramate was dissolved in DMSO at 1:4 (w/v) and diluted with saline prior to injection. Control mice were sham-injected with DMSO diluted in saline. Two days after induction of diabetes, topiramate was administered by daily subcutaneous (sc) injection at 1.0 mg/kg/day. After 10 weeks of treatment, mice were tested in T-maze foot shock avoidance. At the end of 12 weeks of treatment, mice were anesthetized with i.p. injection of urethane (4.0 g/kg). The vascular spaces of the brains were washed free of their contents in preparation for measurement of oxidative stress. The brains were then removed and either stored in an antioxidant buffer [8.6 mM Na2HPO4, 26.6 mM NaH_2_PO_4_, 50 µM butylhydroxy-toluene (BHT), 10 mM aminotriazole, 0.1 mM diethyltri aminepentaacetic acid (DETAPAC)] or at −80°C for later use.

For pericyte dropout studies, at the end of 4-, 8- or 12-week time points, brains were removed and stored at −80°C until isolation of microvessels.

## Measurement of Oxidative Stress in the Brain

### Determination of Glutathione

Reduced glutathione (GSH) concentrations in the brain were determined by reverse phase HPLC as described previously [8]. The HPLC system (Thermo Electron Corporation, Thermo Scientific, Waltham, MS, USA) consisted of a Finnigan Spectra System vacuum membrane degasser (model SCM1000), a gradient pump (model P2000), an autosampler (model AS3000), and a fluorescence detector (model FL3000) with λ_ex_=330 nm and λ_em_=376 nm. The HPLC column used was a Reliasil ODS-1 C_18_ column (5-µm packing material) with 250 mm × 4.6 mm i.d. (Column Engineering, Ontario, CA, USA). The mobile phase (70% acetonitrile and 30% water) was adjusted to pH 2.0 through the addition of 1 ml/L of both acetic and o-phosphoric acids (Fisher Scientific, Fair Lawn, NJ). Brains were homogenized in a serine-borate buffer (100 mM Tris-HCl, 10 mM boric acid, 5 mM L-serine, 1 mM DETAPAC, pH 7.4) on ice. Homogenates were derivatized with 1.0 mM NPM [N-(1-pyrenyl)-maleimide] in acetonitrile. HPLC grade water was added to each sample to make a volume of 250 µl, and 750 µl NPM (1 mM in acetonitrile) was added. This mixture was incubated for 5 min at room temperature and the reaction stopped by adding 10 µl of 2 N HCl. The samples were then filtered through a 0.45-µm acrodisc filter (Advantec MFS, Inc., Dublin, CA, USA) and injected into the HPLC system. The NPM derivatives were eluted from the column isocratically at a flow rate of 1 ml/min.

### Determination of Lipid Peroxidation

Mouse brains were homogenized in Triton X-100 lysis buffer [0.5% Triton X-100, 20 mM Tris (pH 7.4), 0.15 M NaCl, 2 mM EDTA, 1 mM EGTA, and a protease inhibitor cocktail (P2714)], centrifuged at 1,000 × g for 10 min, and supernatants agitated for 30 min. Following centrifugation at 20,000 × g for 40 min, final supernatants were used for detection of 4-hydroxy-2-trans-nonenal (HNE, byproduct of lipid peroxidation) by immunoblots as described previously [8,20]. The proteins (25 µg) were electrophoresed on 4–12% Bis-Tris reducing gels. After transfer to nitrocellulose membranes (0.45 µm pore size), membranes were incubated with anti-HNE antibody (1:2,000) overnight at 4°C and horseradish peroxidase-conjugated secondary antibody (1:10,000) for 1 hour at room temperature. The polypeptides were visualized by chemiluminescent substrate. The membranes were re-probed with anti-γ-tubulin antibody to validate the loading efficiency and the bands were quantified by ImageJ analysis software (Research Services Branch, National Institutes of Mental Health, Bethesda, MD, USA). Optical density values of individual HNE bands within each lane were added to obtain a total value.

### Determination of Pericyte/Endothelial Cell Ratios in the Brain

Mouse brains were harvested from diabetic mice after 4-, 8-, or 12-weeks of topiramate treatment. The microvessels were isolated as described previously [8]. The isolated microvessels were put on two-well chamber slides and air dried. The microvessels were then fixed with 3% paraformaldehyde in PBS for 45 min at room temperature before the slides were stained with Periodic acid-Schiff (PAS)-hematoxylin. This staining was used to analyze pericyte dropout by light microscopy [8]. Captured PAS-stained images of the microvessels were examined by an experienced blinded observer to count the BEC and pericyte nuclei in the same microscope field. Intraluminal cell nuclei with a large oval shape were ascribed as BEC. Typically, cell nuclei placed laterally on the capillary wall (between the two blades of microvascular basal lamina) with a small round shape are pericytes. Segmented nuclei or nuclei without contact with the basal lamina were excluded. Both pericyte and BEC were counted in at least five randomly selected microscopic fields of 100 cells each. Results are presented as the percent of total cells that are pericytes.

### Left-Right T-Maze Foot Shock Active Avoidance Training

We have previously used the T-maze foot shock avoidance apparatus training and testing procedures [21,26], which is a hippocampal task that requires the integration of multiple cues from the environment to solve the task. In this test, we have shown that streptozotocin induced diabetic mice have a poor ability to learn and retain memories [21]. We have previously shown that permanent and temporary damage to as little as 30% of the hippocampus impairs learning and memory in this task [26]. The maze consists of a black plastic start alley with a start box at one end and two goal boxes at the other. A stainless steel rod floor runs throughout the maze. The start box is separated from the start alley by a plastic guillotine door that prevents the mouse from moving down the alley until the training starts.

A training trial begins when a mouse is placed into the start box. When the guillotine door is raised, the buzzer sounds simultaneously and after 5 sec, foot shock is applied. The goal box the mouse first enters on the initial trial is designated as “incorrect.” Foot shock continues until the mouse enters the other goal box, which on all subsequent trials, is designated “correct” for that particular mouse. At the end of each trial, the mouse is removed from the goal box and returned to its home cage. A new trial begins by placing the mouse in the start box, sounding the buzzer, and raising the guillotine door. Foot shock is applied 5 sec later if the mouse does not leave the start box or fails to enter the correct goal box. Training is continued until the mouse makes one avoidance. Retention is tested one week later by continuing the training until each mouse makes 5 avoidances in 6 consecutive training trials. The maximum duration of shock is 30 sec. If a mouse does not escape the shock within 30 sec in the first trial, the mouse is removed from the study. We have found this to occur in less than 1% of our subjects.

The training condition uses 45 sec inter-trial intervals with the conditioned stimulus at 65 dB and foot shock set at 0.40 mA on a scrambled grid floor shocker (Model E13-08, Coulbourn Instruments, Whitehall, PA, USA).

### Statistical Analysis

All means are reported with their n and SEM. Two means were compared by the unpaired two-tailed Student’s t test. For more than two means, analysis of variance (ANOVA) followed by Newman-Keuls multiple comparison test was used. When there were two independent variables, a two-way ANOVA, followed by a multiple group comparison test (Bonferroni), was used to assess statistical significance. The measure of acquisition was the number of trials to first avoidance, and retention in the T-maze was the number of trials to reach the criterion of 5 avoidances in 6 consecutive trials. Tukey’s post-hoc analysis was used to compare means between groups. Results were considered significant at p<0.05. Statistical analyses were made using the GraphPad Prism 5.0 package program (GraphPad Software Inc., San Diego, CA, USA).

## Results

### Effect of Topiramate Treatment on Oxidative Stress in the Mouse Brain

**Figure 1:**
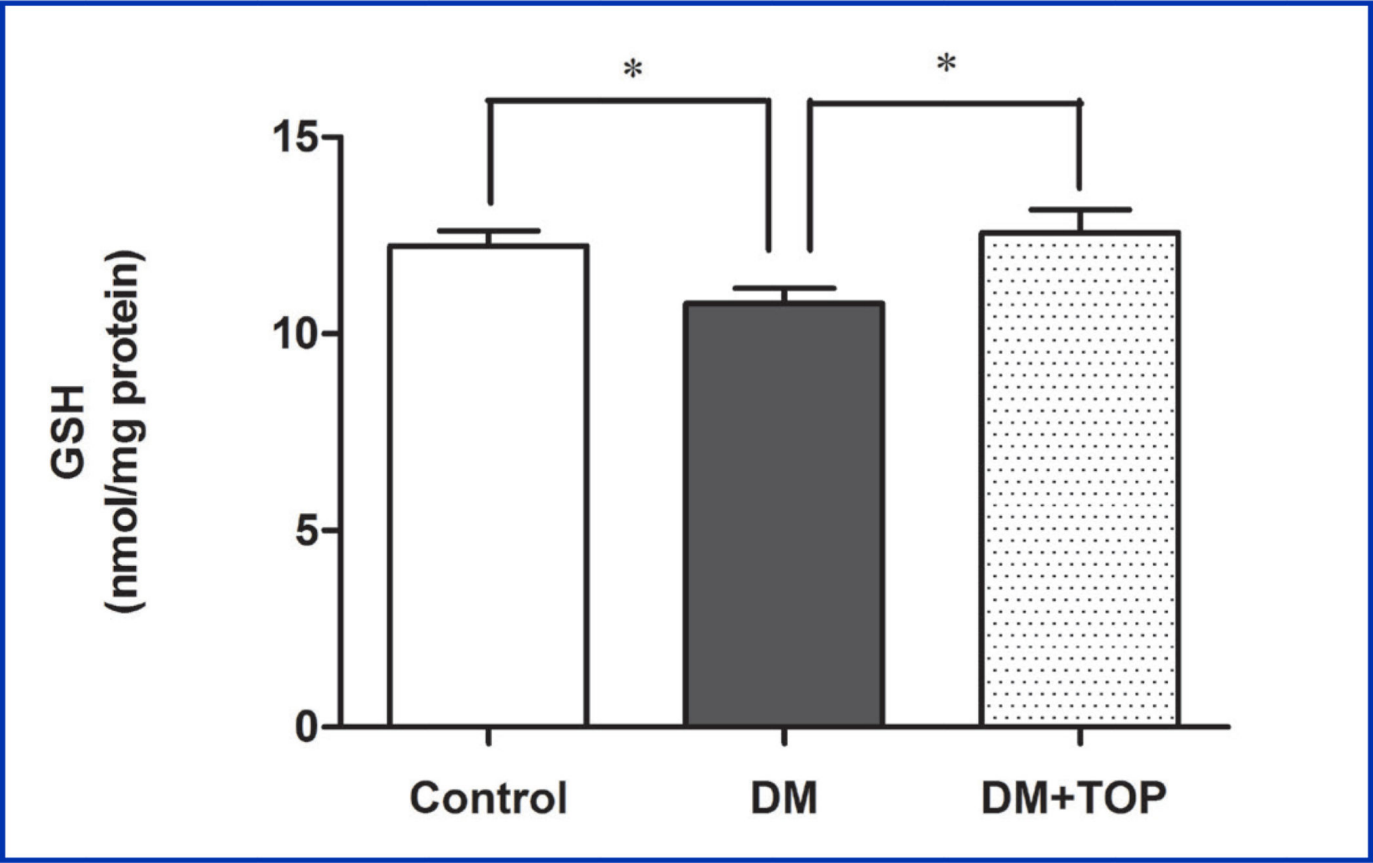
Effect of topiramate on diabetes-induced decline in GSH levels in the mouse brain. Two days after induction of diabetes, mice were given daily sc injections of topiramate for 12 weeks and brains were analyzed for GSH levels by HPLC. The significantly lower GSH levels in the diabetic animals (DM) were restored to control levels by topiramate treatment (DM+TOP). The values are expressed as mean ± SEM (n=8–10/group) (*p<0.05).

A significant decrease in the GSH content (DM, 10.8 ± 0.4 vs. control, 12.2 ± 0.4, p<0.05) was found in the brain of diabetic mice (Fig. 1 and Table 1) compared to the controls, indicating oxidative stress. Topiramate treatment significantly increased the levels of GSH in these mice (DM+TOP, 12.6 ± 0.6, vs. DM, 10.8 ± 0.4, p<0.05).

**Figure 2:**
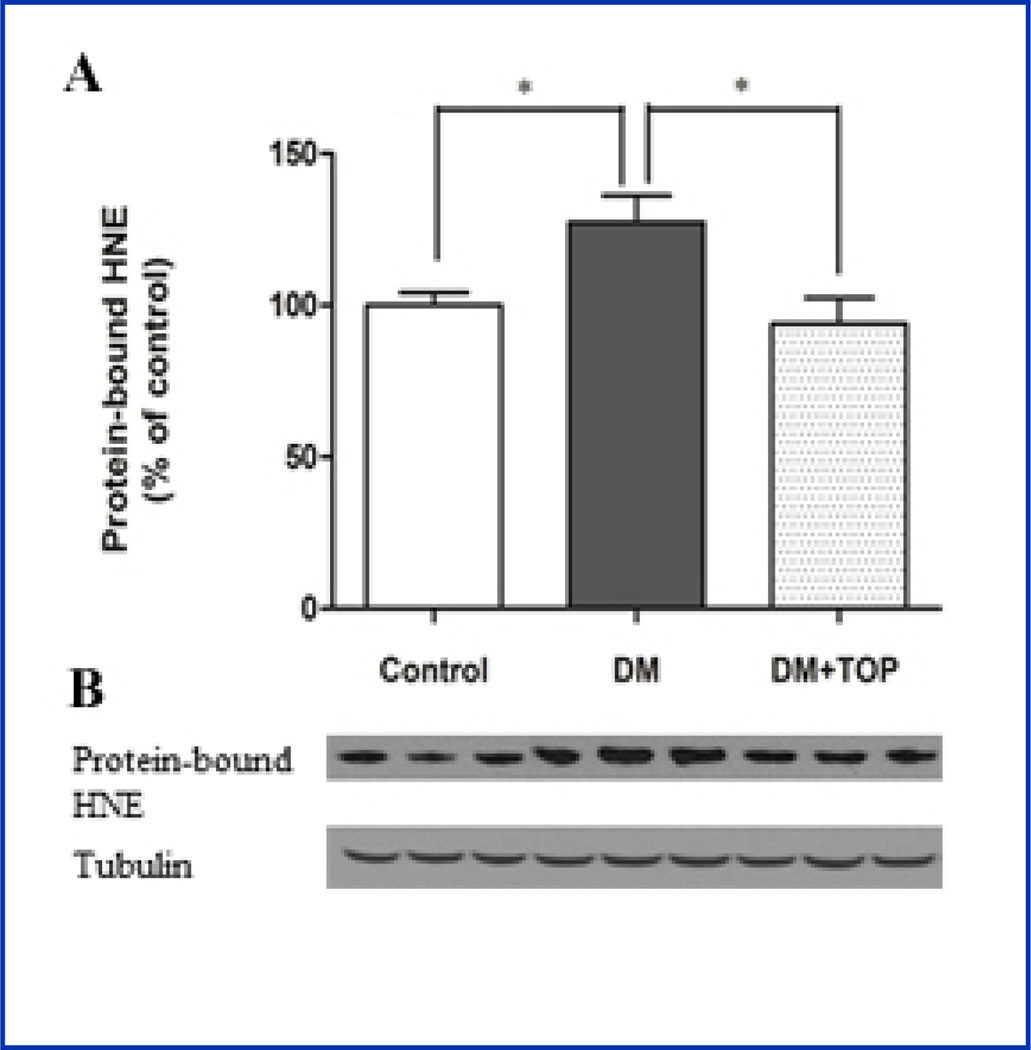
Effect of topiramate on diabetes-induced lipid peroxidation in the mouse brain. Two days after induction of diabetes, mice were given daily sc injections of topiramate for 12 weeks, and brains were analyzed for HNE by immunoblot analysis. **A**, The significantly high levels of HNE in diabetic mice (DM) were reversed in topiramate-treated (DM+TOP) mice. **B**, Representative immunoblot of protein-bound HNE. The values are expressed as mean ± SEM (n=8–10/group) (*p<0.05).

Lipid peroxidation, another measure of oxidative stress, was assessed by analyzing the levels of protein-bound HNE, a stable byproduct of lipid peroxidation. Higher levels of HNE signify oxidative stress. A significant elevation in HNE-bound proteins was observed in diabetic mice (DM, 127.3 ± 8.8 vs. control, 100 ± 4.2, p<0.05), compared to control mice (Fig. 2 and Table 2). Topiramate significantly reduced the protein bound HNE (DM+TOP, 93.9 ± 8.4 vs. DM, 127.3 ± 8.8, p<0.05) in treated diabetic mice.

### Effect of Topiramate Treatment on Pericyte/Endothelial Cell Ratios

**Figure 3:**
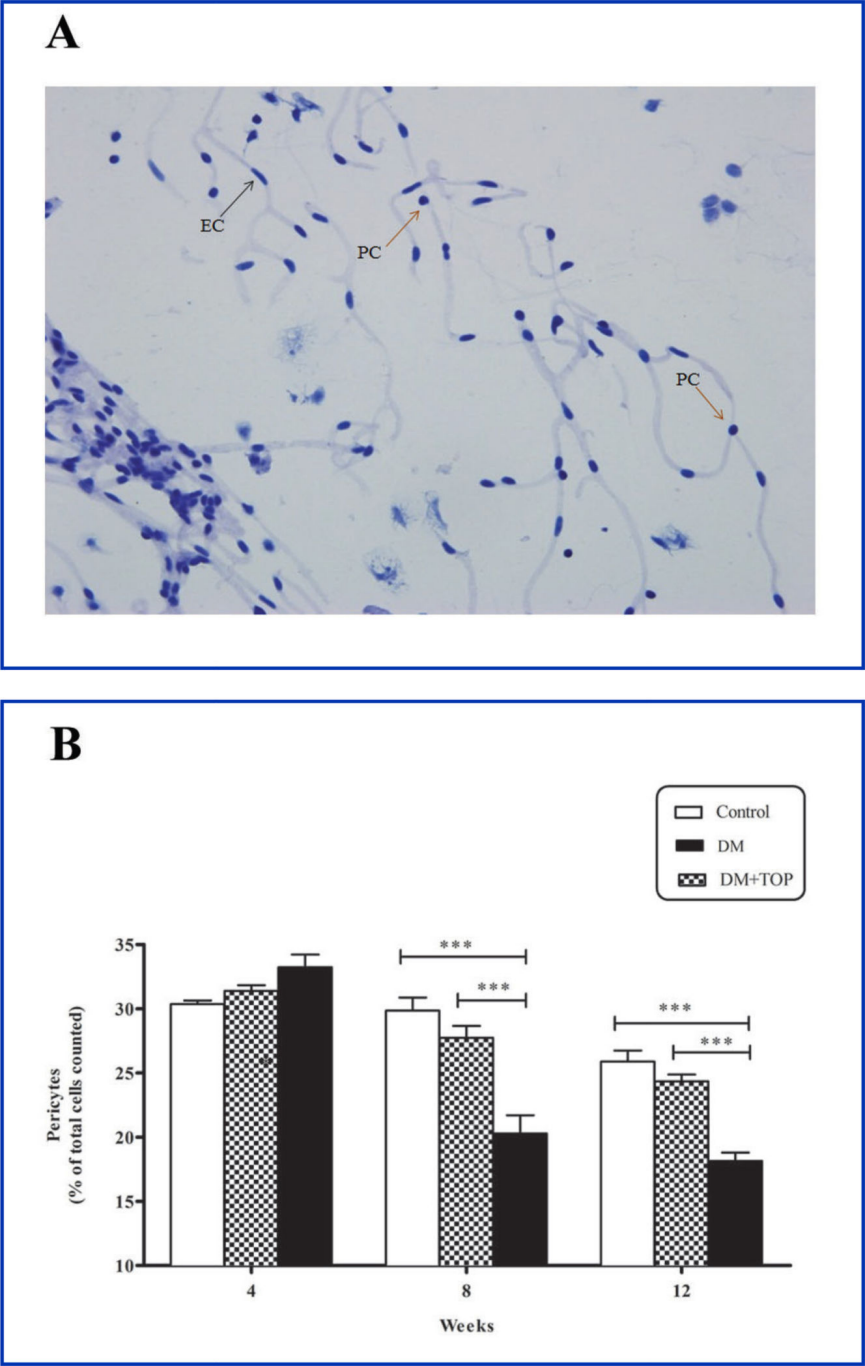
Effect of topiramate on diabetes-induced decline in pericyte/BEC ratios in cerebral microvasculature. Diabetic mice were given daily sc injections of topiramate for up to 12 weeks. Brains were harvested at 4-, 8-, and 12-weeks of topiramate treatment, and cerebral microvessels were isolated and stained with PAS-hematoxylin on chamber slides. The pericytes and BEC, in the isolated microvessels, were counted, and the percent of pericytes/total cells was calculated. **A**, Captured PAS-hemotoxylin-stained microscope image of isolated mouse brain microvessels. Arrows point to prominent round nuclei of pericytes (PC), and to elongated cigar shaped nucleus of endothelial cell (EC). **B**, After an initial non-significant increase in pericyte numbers in diabetic mice (DM) at 4 weeks of diabetes, the numbers declined significantly below the controls at 8 and 12 weeks. The pericyte numbers were significantly higher at both 8 and 12 weeks of diabetes in topiramate-treated mice (DM+TOP). The values are expressed as mean ± SEM (n=10/group) (***p<0.001).

The numbers of pericytes and BEC were determined in PAS-stained, isolated brain microvessels (Fig. 3 and Table 3). An initial, non-significant increase in pericyte numbers was observed in both diabetic mice (DM, 33.23 ± 0.99 vs. control, 30.36 ± 0.28; ns) and topiramate-treated diabetic mice (DM+TOP, 31.39 ± 0.44 vs. control, 30.36 ± 0.28; ns) at 4 weeks of diabetes. Thereafter, pericyte numbers significantly decreased at 8 weeks (DM, 20.28 ± 1.43 vs. control, 29.85 ± 1.01, p<0.001) and 12 weeks (DM, 18.14 ± 0.66 vs. control, 25.89 ± 0.85, p<0.001) in diabetic mice compared to control (Fig. 3 and Table 3)

As expected, topiramate treatment rescued pericyte loss and the number of pericytes at both 8 weeks (DM+TOP, 27.73 ± 0.93 vs. DM, 20.28 ± 1.43, p<0.001) and 12 weeks (DM+TOP, 24.35 ± 0.53 vs. DM, 18.14 ± 0.66, p<0.001) was significantly higher than in diabetic mice. The number of pericytes in topiramate-treated diabetic mice was essentially the same as non-diabetic controls at 8 weeks (DM+TOP, 27.73 ± 0.93 vs. control, 29.85 ± 1.01, p<0.001) and 12 weeks (DM+TOP, 24.35 ± 0.53 vs. control, 25.89 ± 0.85, p<0.001).

### The Effect of Topiramate on T-maze Foot Shock Avoidance

**Figure 4:**
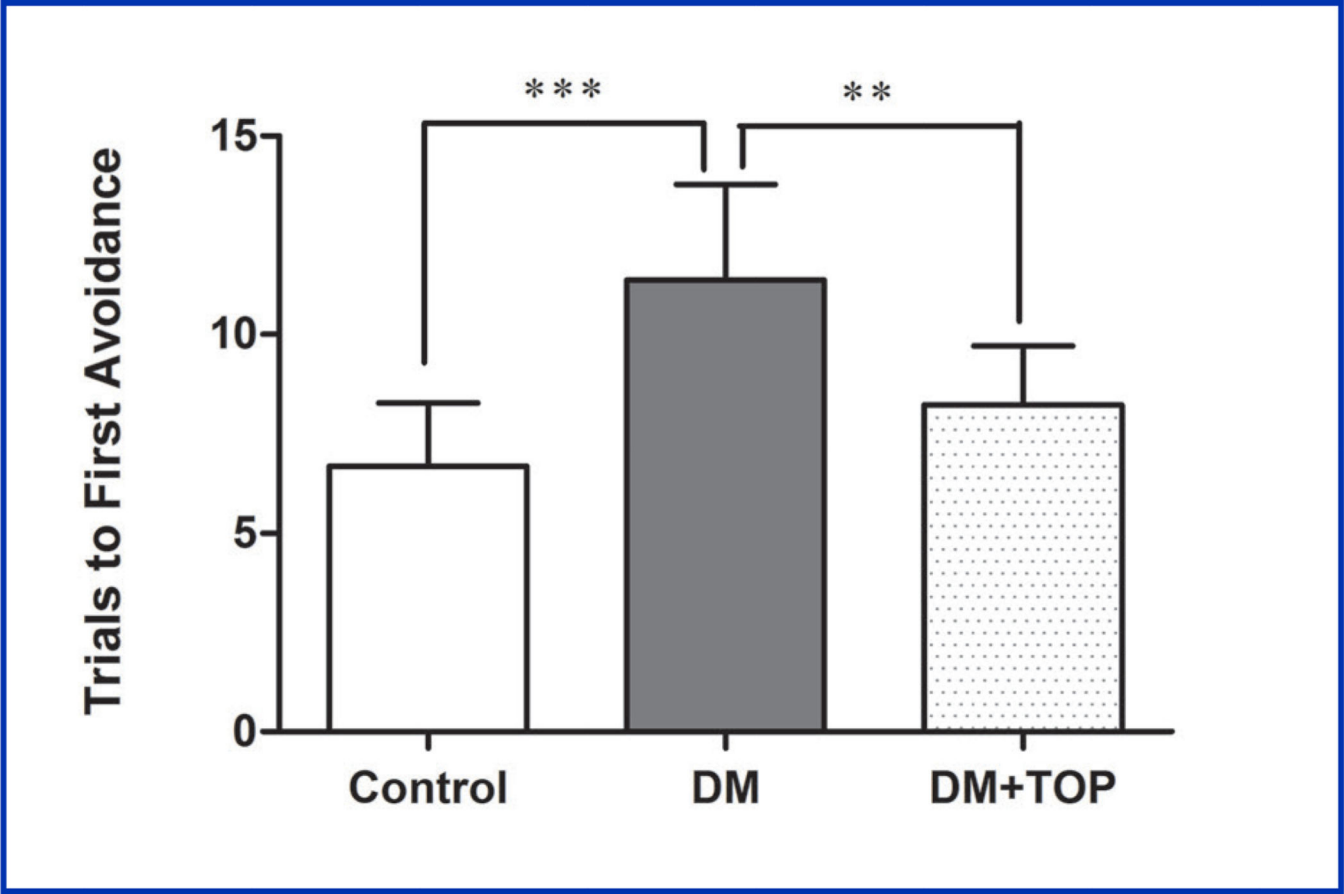
Effect of topiramate on the performance of T-maze acquisition. Two days after induction of diabetes, mice were given daily sc injections of topiramate for 12 weeks. The animals were analyzed for learning behavior by T-maze foot shock avoidance test. A significant decline in learning behavior was observed in diabetic mice (DM). Topiramate treatment led to improved learning in treated diabetic mice (DM+TOP). The values are expressed as mean ± SEM (n=8–10/group) (**p<0.01, ***p<0.001).

A two-way ANOVA with trials to first avoidance as the independent variable showed a significant effect for diabetic state F (1,55) = 25.10, p<0.01. Diabetic mice took a significantly greater number of trials to reach first avoidance than the non-diabetic mice (Fig. 4 and Table 4). Tukey’s post-hoc analysis indicated that diabetic mice that received 1.0 mg/kg topiramate took significantly fewer trials to reach first avoidance than the diabetic mice (Fig. 4 and Table 4). The two-way ANOVA for trials to criterion on the retention test showed a significant effect for diabetic state F (1,54) = 15.56, p<0.002. Tukey’s post-hoc analysis indicated that there were no significant differences between groups (data not shown).

## Discussion

We previously reported a decrease in pericyte numbers in the diabetic mouse brain caused by hyperglycemia-induced oxidative stress. We also showed that treatment with topiramate reduced the oxidative stress and restored pericyte numbers [8]. In the published studies, a dose of 50 mg/kg topiramate was used, which is equivalent to the 200 mg/day topiramate dosage currently in clinical use for reducing alcohol consumption and epileptic seizures [31,32]. Unfortunately, this dose of topiramate has serious side effects, such as psychomotor slowing, difficulty with concentration, speech and language problems, and somnolence and fatigue [32].

The preventative effects of topiramate on epileptic seizures are due to the ability of the drug to modulate voltage-dependent sodium conductance, to potentiate gamma-amino butyric acid-evoked currents, and to block the kainite/AMPA (α-amino-3-hydroxy-5-methylisoxazole-4-propionic acid) subtype of the glutamate receptor [27]. The ability to stop alcohol abuse is attributed to the potential of topiramate to decrease mesocorticolimbic dopamine activity after alcohol intake and to antagonize the chronic change induced by alcohol at the kainite/AMPA glutamate receptor [31]. Whereas our published data show [8,20,33] that the effect of topiramate in reducing oxidative stress and pericyte loss is due to the ability of the drug to inhibit mitochondrial carbonic anhydrases (CAs).

Hyperglycemia-induced oxidative stress is caused by overproduction of reactive oxygen species (ROS) primarily during respiration (mitochondrial oxidative metabolism of glucose). Rate of respiration is regulated by mitochondrial CAs [8,20,33]. Briefly, glucose is metabolized in the cytosol to pyruvate in the cytoplasm. Pyruvate enters the mitochondria where it combines with bicarbonate (HCO_3_^−^) to make oxaloacetate, a key enzyme in the oxidative metabolism of glucose. The HCO_3_^−^ required for carboxylation of pyruvate has to be produced inside the mitochondria. It cannot be imported from the cytosol because mitochondrial membranes are impermeant to HCO_3_^−^. Mitochondrial CAs, the carbonic anhydrases inside the mitochondria, provide HCO_3_^−^ by reversible hydration of carbon dioxide (CO_2_ + H_2_O ⇔ HCO_3_^−^ + H^+^). Oxaloacetate, upon entering the Krebs cycle, generates electron donors, FADH_2_ and NADH, which are carried to electron transport chain reactions (ETC) to generate ATP. Superoxide, the precursor of all ROS [34] is produced as a byproduct of ETC reactions. In diabetes, constant influx of glucose in relatively insulin-insensitive tissues such as brain, eye, nerves, etc., causes an overproduction of superoxide as follows: The excess electron donors produced during the Krebs cycle generate high mitochondrial membrane potential by pumping protons across the inner mitochondrial membrane. The high mitochondrial membrane potential inhibits electron transport at complex III, increases the half-life of the free radical intermediate of coenzyme Q, which reduces O_2_ to superoxide, leading to production of ROS above physiological levels. Though small fluctuations in the steady-state concentration of these oxidants may actually play a role in intracellular signaling [35], uncontrolled increases in the steady-state concentrations of these oxidants lead to free radical mediated chain reactions which indiscriminately target proteins [36], lipids [37], polysaccharides [38], and DNA [39,40], and result in oxidative stress. Our published data show that pharmacological inhibition of mitochondrial CAs slows the rate of respiration, ROS production, and oxidative stress in brain pericytes [20,33]. We have also reported that mice in which mitochondrial CAs have been genetically knocked out [29] exhibit reduced oxidative stress in the brain [8], and topiramate, a mitochondrial CA inhibitor, protects the brain from oxidative stress and pericyte loss in vivo [8]. Our published data also show that, in culture, the high glucose-induced intracellular oxidative stress and apoptosis of cerebral pericytes are significantly reduced by treatment with topiramate [20].

Since topiramate has high affinity for both CAVA (Ki, 63 nM) and CAVB (Ki, 30 nM), it is conceivable that a significantly lower dose of the drug will inhibit mitochondrial CAs [41] and effectively reduce the oxidative stress and consequent brain injury. Therefore, the current study was designed to determine a minimum effective dose of topiramate that can protect the brain from diabetic injury. Since diabetes is associated with its own pattern of cognitive impairment [42–44], in addition to being a risk factor for stroke and Alzheimer’s disease, a dose of 1.0 mg/kg topiramate was used in the current study based on its effectiveness in consolidation and retrieval recognition memory in rats [45]. We now report that the treatment of diabetic mice with topiramate at this low dose reduces oxidative stress (Fig. 1,2, and Table 1 and 2) and restores pericyte numbers (Fig. 3 and Table 3) in the brain, as well as corrects impaired learning behavior (Fig. 4 and Table 4) caused by diabetes. Memory decline associated with diabetes did not improve with topiramate treatment. This result may be due to the fact that, in addition to oxidative stress, other factors are contributing to memory deficit in diabetes.

A dose of 1.0 mg/kg topiramate, which protects the mouse brain from diabetic damage, translates to 5.0 mg/day for a 60 kg human being [28]. These results suggest that topiramate at 5.0 mg/day can help slow the onset and delay the progression of diabetic damage to the human brain [5,7]. Since similar events occur the capillary beds of nerves, another insulin-insensitive tissue [46], topiramate may also help treat peripheral neuropathy caused by diabetes [47].

Topiramate has not yet been tested for the treatment of hyperglycemia-induced oxidative damage to the diabetic brain. The maintenance of good glycemic control remains as one of the most effective options to prevent or delay the progression of the disease. However, good glycemic control for most is difficult to achieve and even harder to maintain. We recommend inclusion of 5.0 mg/daily topiramate for both type 1 and type 2 diabetic patients along with glycemic control drugs.

Clinical trials with this least toxic dose of topiramate are a distinct possibility since the drug has been in clinical use for a long time for prevention of epileptic seizures [32], for treatment of alcohol dependency [31], and obesity [48,49]. More recently, topiramate in combination with phentermine (Qsymia) has been approved by FDA for weight loss.

## Figures and Tables

**Table 1:** Effect of topiramate on diabetes-induced decline in GSH levels in the mouse brain.

Groups	GSH (nmol/mg protein) Mean ±SEM @ 95 % CI	ANOVA P-Value
**Control**	12.2 ± 0.4	<0.05
**DM**	10.8 ± 0.4*	
**DM+TOP**	12.6 ± 0.6	

Values are mean ± SEM (ANOVA, Bonferroni), n = 8–10/group.

* p<0.05 for difference from DM+TOP and control group.

**Table 2:** Effect of topiramate on diabetes-induced lipid peroxidation in the mouse brain.

Groups	Protein-bound HNE (% of control) Mean ± SEM @ 95 % CI	ANOVA P- Value
**Control**	100 ± 4.2	<0.05
**DM**	127.3 ± 8.8*	
**DM+TOP**	93.9 ± 8.4	

Values are mean ± SEM (ANOVA, Bonferroni), n = 8–10/group.

* p<0.05 for difference from DM+TOP and control group.

**Table 3:** Effect of topiramate on diabetes-induced decline in pericyte/BEC ratios in cerebral microvasculature.

Groups	4 weeks Mean ± SEM @ 95% CI	8 weeks Mean ± SEM @ 95% CI	12 weeks Mean ± SEM @ 95% CI	ANOVA P- Value
**Control**	30.36 ± 0.28	29.85 ± 1.02	25.89 ± 0.85	<0.001
**DM**	33.23 ± 0.99	20.28 ± 1.43	18.14 ± 0.66	
**DM+TOP**	31.39 ± 0.44	27.73 ± 0.93***	24.35 ± 0.53***	

Values are mean ± SEM (ANOVA, Bonferroni), n = 10/group.

*** p<0.001 for difference from DM and control groups.

**Table 4:** Effect of topiramate on the performance of T-maze acquisition.

Groups	Trials to First Avoidance Mean ± SEM @ 95 % CI	ANOVA P- Value
**Control**	6.7 ± 0.5	<0.01
**DM**	11.4 ± 0.7***	
**DM+TOP**	8.2 ± 0.5**	

Values are mean ± SEM (ANOVA, Tukey’s post-hoc), n = 8–10/group.

** p<0.01 for difference from DM and

*** p<0.001 for difference from control.
